# Negative Samples for Improving Object Detection—A Case Study in AI-Assisted Colonoscopy for Polyp Detection

**DOI:** 10.3390/diagnostics13050966

**Published:** 2023-03-03

**Authors:** Alba Nogueira-Rodríguez, Daniel Glez-Peña, Miguel Reboiro-Jato, Hugo López-Fernández

**Affiliations:** 1 CINBIO, Department of Computer Science, ESEI—Escuela Superior de Ingeniería Informática, Universidade de Vigo, 32004 Ourense, Spain; 2 SING Research Group, Galicia Sur Health Research Institute (IIS Galicia Sur), SERGAS-UVIGO, 36213 Vigo, Spain

**Keywords:** colorectal cancer, deep learning, convolutional neural network (CNN), polyp detection, polyp localization

## Abstract

Deep learning object-detection models are being successfully applied to develop computer-aided diagnosis systems for aiding polyp detection during colonoscopies. Here, we evidence the need to include negative samples for both (i) reducing false positives during the polyp-finding phase, by including images with artifacts that may confuse the detection models (e.g., medical instruments, water jets, feces, blood, excessive proximity of the camera to the colon wall, blurred images, etc.) that are usually not included in model development datasets, and (ii) correctly estimating a more realistic performance of the models. By retraining our previously developed YOLOv3-based detection model with a dataset that includes 15% of additional not-polyp images with a variety of artifacts, we were able to generally improve its F1 performance in our internal test datasets (from an average F1 of 0.869 to 0.893), which now include such type of images, as well as in four public datasets that include not-polyp images (from an average F1 of 0.695 to 0.722).

## 1. Introduction

Computer-aided diagnosis (CAD) systems for polyp detection are now reality, thanks to the advances in and the application of deep learning (DL). So far, many randomized control trials (RCT) have already been performed [1,2,3,4,5,6], some of them associated with commercial systems [7], and many more are being developed. The growing number of reviews on this topic demonstrates that polyp detection is much more advanced than polyp characterization [5,7,8,9], and a recent meta-analysis of six RCTs showed that there is an increase in both adenoma and polyp detection rates with the utilization of artificial intelligence (AI)-assisted colonoscopy [5].

In the context of the PolyDeep project (https://www.polydeep.org, accessed on 11 January 2023), we developed and reported, in 2021, a real-time polyp detection model based on a YOLOv3 pre-trained with PASCAL VOC datasets [10]. The base model was fine-tuned using a private dataset containing 941 different polyps and 28,576 images annotated by expert endoscopists [11]. The performance of this model in a bounding box-based evaluation using still images (a test partition of our private dataset) was an F1 score of 0.88 (recall = 0.87, precision = 0.89), reaching a state-of-the-art level.

In follow-up work [12], we reported, in 2022, the results of testing this model on ten public colonoscopy image datasets, which, to the best of our knowledge, was the biggest systematic evaluation of a polyp detection model without retraining. We also analyzed these results in the context of the results from another 20 state-of-the-art publications using the same public datasets. We observed that our model had an average F1 score decay of 13.65% when tested on the ten public datasets. This number may seem high, but it must be taken into account the high degree of variability in the public datasets, as some of them contain not-polyp images and multiple-polyp images, resulting in a slightly different scenario compared to the type of images used to develop our base model (only polyp-containing images).

Since then, we have also released an updated version of our dataset and other complementary datasets through the PIBAdb Colorectal Polyp Image Cohort (https://www.iisgaliciasur.es/home/biobanco/cohorte-pibadb, accessed on 11 January 2023), which is publicly available at the biobank of the Galicia Sur Health Research Institute (IISGS). This image cohort was created with the aim of making public the data collected during the PolyDeep project, including, mainly, images with polyps located with bounding boxes annotated by expert endoscopists, but also other colon images without polyps and the videos of colonoscopies from which the images were extracted. Notably, one of the complementary datasets published includes ~14,000 not-polyp images (the “PIBAdb Not Polyp” image gallery). These images without polyps have been extracted automatically (one image per second) from annotated videos and they may contain artifacts (e.g., instrumental, on-screen information, etc.), be blurred or very close to mucosa. This follows the general trend of the latest public datasets available, which are larger and include images without polyps.

In light of the findings of our previous works, our efforts to keep improving our model have been data-centric. In this regard, some studies have already used not-polyp images during training to improve the performance of the model [13], and, therefore, in this work we assess the usefulness of the recent not-polyp images included in the PIBAdb database to improve the performance of our previous model. Our belief is that training using these images will allow us to improve the predictive positive value of the model (precision), while maintaining its sensitivity levels (recall).

### Rationale (Hypothesis)

The rationale behind the inclusion of no-object images, or negative samples, in an object detection network is guided by the fact that, when such models are implemented in a real setting, the model could face out-of-distribution samples, that is, image types that have content that was not used during the model development.

A polyp detection network trained only with polyp-containing images (at least one polyp per image) uses the outside-boxes image content as the “negative” samples, learning not to predict bounding boxes there. This may be sufficient if all the possible negative content is there, but this is not the case in endoscopies, especially during the polyp-finding phase. The normal mucosa content behind a polyp is not the only negative content to learn from. There are many other scenarios (out-of-distribution) that will arise during the exploration (e.g., medical instruments, water jets, feces, blood, excessive proximity of the camera to the colon wall, blurred images, etc.) that could lead the network to identify parts of the image as something similar to a polyp, or, at least, more similar to a polyp than to normal mucosa, producing many false positives.

With this study, we aim at exploiting this hypothesis by retraining our model with such out-of-distribution images in order to improve its positive predictive value, while maintaining its sensitivity. Moreover, we will put special focus on testing the model with this kind of image at different proportions, since test datasets that do not include them are not able to capture the real performance of detection models in the real setting. In summary, we want to move out-of-distribution samples to in-distribution samples.

## 2. Materials and Methods

### 2.1. Our Base Model for Polyp Detection

In 2021, we reported the results of a real-time colorectal polyp detection model based on YOLOv3 [11]. This model was trained and evaluated with a private dataset built using 28,576 images with located polyps from the PIBAdb cohort, both with white light (WL) and narrow-band imaging (NBI) light. This dataset includes a subset of images automatically extracted at a rate of one image per second (16,691) and a subset of images manually selected by expert endoscopists (11,885).

For the model development, the private dataset was divided into three partitions: (i) a training partition (49%; 13,873 images) for model training during the development phase, (ii) a validation partition (21%; 6045 images) for model evaluation and selecting the best model during the development phase, and (iii) a test partition (30%; 8658 images) for best model evaluation after the development phase. The resulting model, pre-trained with the PASCAL VOC 2007 and 2012 challenges and fine-tuned with the dataset comprising both the training and validation partitions, achieved an F1 score of 0.88 (recall = 0.87, precision = 0.89) and an average precision (AP) of 0.87 evaluated on the test partition. As noted in the introduction, this model was later evaluated on ten public datasets, without retraining, in order to assess its generalization capabilities [12], showing an average F1 decay of 13.65%.

### 2.2. Public Datasets

Table 1 shows the most relevant details of the ten public colonoscopy image datasets selected in our previous study [12], which are also the subject of this study. Regarding polyp location in the dataset images, four dataset provide it as bounding boxes (KUMC [14], SUN [13], LDPolypVideo [15], and Kvasir-SEG [16]), while seven datasets use binary masks (CVC-ClinicDB [17], CVC-ColonDB [18,19], CVC-PolypHD [18,19], ETIS-Larib [20], CVC-ClinicVideoDB [21,22], PICCOLO [23], and Kvasir-SEG [16]). As the polyp detection model evaluated in this work uses bounding boxes, it was necessary to transform the binary masks into bounding boxes in six datasets (ClinicDB [17], CVC-ColonDB [18,19], CVC-PolypHD [18,19], ETIS-Larib [20], CVC-ClinicVideoDB [21,22], and PICCOLO [23]. The process followed to perform these transformations was explained in our previous study [12], and the conversion scripts are available at https://github.com/sing-group/public-datasets-to-voc (accessed on 11 January 2023).

Taking into account the characteristics of the different datasets, a high degree of variability can be observed with respect to the number of images included, and whether they contain not-polyp images or not [8,24]. Nevertheless, the trend in recent years is to create increasingly larger datasets with the presence of images without polyps.

### 2.3. Experimental Setup

The experiments consist of training new models using the original polyp image dataset of our base model and progressively injecting it with different percentages of not-polyp images. The original dataset partitions (i.e., training, validation, and test) were augmented with not-polyp images at rates around 2%, 5%, 10%, and 15% of the initial number of images in the partition.

**Figure 1:**
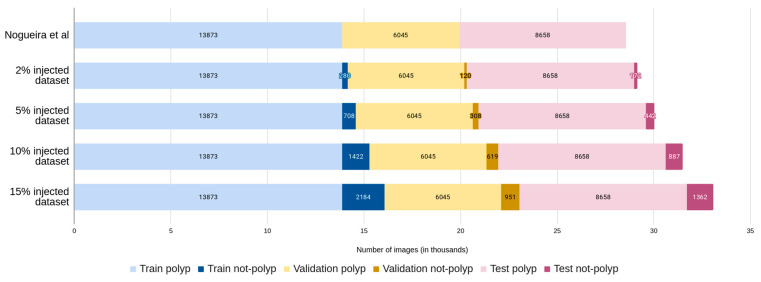
Not-polyp datasets created from the original polyp images dataset [11].

Starting from the images of each original partition, where each polyp only belongs to one single partition, we progressively added each percentage of images without polyps. The image injection was performed incrementally for each percentage, ensuring that the same not-polyp image was in the same partition type (training, validation, or test) and that the images found in the lower percentage datasets were part of the higher percentage datasets, creating supersets for each partition and dataset. For example, all the images in the 2% training are also included in the 5% dataset, along with an additional 3% of new not-polyp images. The number of resulting images for each partition is shown in the following, Figure 1.

**Figure 2:**
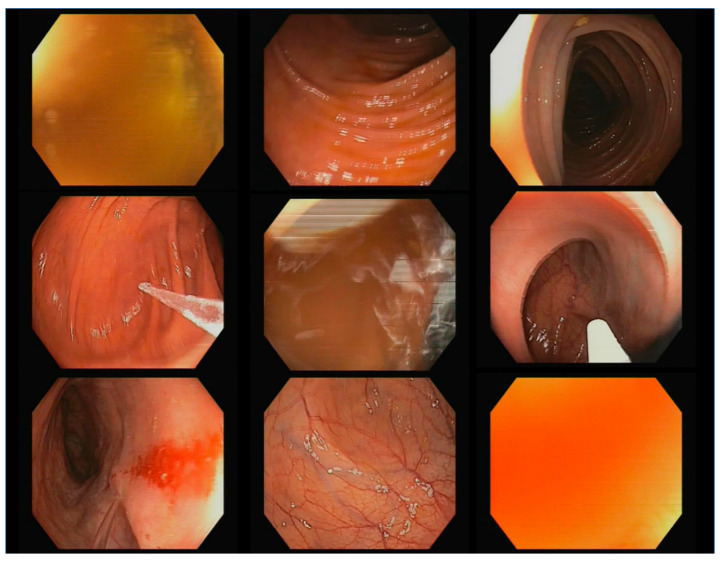
Random sample of the not-polyp images included in the injected datasets.

Figure 2 shows diverse examples of not-polyp images that have been used to train the new networks. As can be seen, these images may contain medical instruments, water jets, feces, blood, excessive proximity of the camera to the colon wall, blurred images, etc.

As in our previous works [11,12], the model development and evaluation was done using Compi [25,26] pipelines, which can be found at the GitHub repository: https://github.com/sing-group/polydeep-object-detection (accessed on 11 January 2023). Specifically, the model training was performed using the train.xml pipeline, while the model evaluation was performed using the text.xml pipeline of this repository.

Once retrained (see details in “Model development and selection”), we evaluated the performance of each new model in an (i) intra-dataset evaluation, using the test partition of the base model, as well as the not-polyp augmented test partitions, and in an (ii) inter-dataset evaluation, using the ten public colonoscopy imaging datasets selected in the previous study.

### 2.4. Evaluation Metrics

Since the main goal of a colorectal polyp detection model is to identify all the polyps shown on camera during a colonoscopy, we have selected recall (or sensitivity) as one of the evaluation measures. In addition, as the ability to not show detections in locations where there is no polyp is also important, because they can disturb or distract an endoscopist, we have also selected precision (or positive predictive value) as a second evaluation measure. Finally, since it acts as a summary of the two evaluation measures mentioned above, we have chosen $F_{1}$ as the main evaluation measure.

Given that the output of the detection model is a bounding box, in this work we define:

- True Positive (TP): as a predicted bounding box that is over a true bounding box.
- False Positive (FP): as a predicted bounding box that is not over a true bounding box.
- False Negative (FN): as a true bounding box where there is no predicted bounding box over it.

Taking into account these definitions, the selected performance metrics (i.e., recall, precision, and $F_{1}$) are defined as follows:

$$\mathrm{recall}\left(\mathrm{or}\;\mathrm{sensitivity}\right)=\frac{\mathrm{TP}}{\mathrm{TP}+\mathrm{FN}}$$

$$\mathrm{precision}\left(\mathrm{or}\;\mathrm{positive}\;\mathrm{predictive}\;\mathrm{value}\right)=\frac{\mathrm{TP}}{\mathrm{TP}+\mathrm{FP}}$$

$$F_{1}=2\times \frac{\mathrm{precision}\cdot \mathrm{recall}}{\mathrm{precision}+\mathrm{recall}}=\frac{2\times \mathrm{TP}}{2\times \mathrm{TP}+\mathrm{FP}+\mathrm{FN}}$$

## 3. Results and Discussion

The experiments are focused on obtaining the performance metrics of each model in a test partition with similar images with which it was trained (intra-dataset evaluation) and, also, evaluating the models with a test partition where the images belong to public datasets (inter-dataset evaluation). The results are completed with a frame analysis to see how false positive identifications are removed.

### 3.1. Model Development and Selection

Following the same strategy as in our previous works [11,12], each model was retrained again, starting from the initial pre-trained YOLOv3 and using the training partition for 50 epochs. The same training parameters were used for all the models. The best model is selected by the maximum AP achieved in the validation partition during those 50 epochs. Once the best model is selected, it is configured with the confidence score threshold that maximizes the F1 score in the validation partition. Table 2 shows the metrics of the selected models with the best AP achieved in the validation partition images during the training process. AP is equivalent to the Area Under the Precision-Recall Curve (AUPRC), being the average precision at various confidence score thresholds.

As a result, we have the original model (“Nogueira et al.”), plus four additional models (“Model injection *p*”, where *p* is the percentage of injection of not-polyp images into the dataset partitions used to develop the model).

### 3.2. Intra-Dataset Evaluation

First, we evaluated the models trained after the injection of not-polyp images using the test partition of the base model (Nogueira et al., i.e., without not-polyp images). The results are shown in Table 3.

Regarding recall, all the models show a slight fluctuation, ranging from 0.85 in the model trained with the addition of 10% not-polyp images, to 0.88 in the model trained with the addition of 15% not-polyp images. On the other hand, the precision increases as the models are trained with increasing numbers of not-polyp images, and thus achieves the highest value in the model trained with the addition of 15% of not-polyp images (0.91), which also obtains the best F1 score (0.895). In comparison with the base model, the models trained with the addition of 5% and 15% not-polyp images show a slightly better F1 score, whereas the 2% and 10% models a slightly lower one. In summary, the difference is small, and no model shows a clear improvement in the original test partition.

However, as we are looking for a test scenario that better reflects the out-of-distribution samples that the models will face in a real setting, mimicked by including not-polyp images in the test partition, we also performed the evaluation with the test partitions that include different proportions of not-polyp images (also from 2% up to 15%). Since the true polyp images remain the same in all the new not-polyp-containing test partitions, the recall of any given model will remain exactly the same as shown in Table 3, but the precision, and thus the F1, are expected to be affected. Concretely, Table 4 shows the different precision metrics obtained by testing all the models with the different test partitions.

As can be seen, precision is positively correlated with the number of not-polyp images used for training the different models, which evidences the benefit of training with not-polyp images, and it is inversely correlated with the number of not-polyp images in the test partition, which is a statistically expected effect.

**Figure 3:**
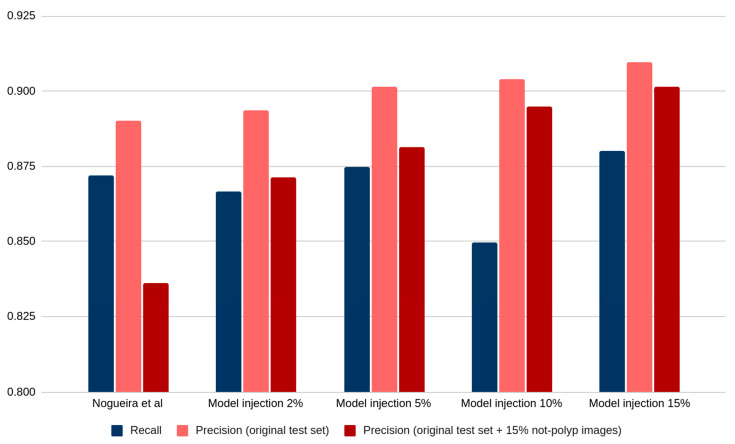
Recall of the five trained models (blue) and their precision in both the original test partition (pink) and the same with the addition of 15% of not-polyp images (dark red) [11].

Putting it all together, Figure 3 shows the recall and precision comparing two distant test partitions: the original one, which does not contain any not-polyp images, and the largest not-polyp containing test partition, which contains the addition of 15% of not-polyp images.

It can be observed from Figure 3 that the precision decay of the original model is the biggest one (red bars in the leftmost column of Figure 3). However, as we create models trained with an increasing number of not-polyp images, this precision recovers (dark red bars’ progression in Figure 3).

**Figure 4:**
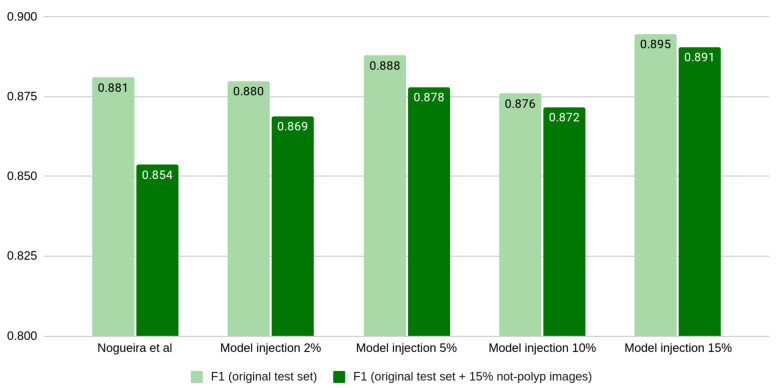
F1 of the five trained models in both the original test partition (light green) and the same with the addition of 15% of not-polyp images (dark green) [11].

In order to compare all the models, Figure 4 shows the F1 score of all the models in both test scenarios (without not-polyp images and with the addition of 15% of not-polyp images). As it can be seen, the test partition that includes the addition of 15% of not-polyp images (dark bars in Figure 4) is more challenging, and each model attains a lower F1 score. Moreover, the addition of not-polyp images for training does not show a consistent benefit in the original test partition (light bars in Figure 4), whereas it does in the test partition that adds 15% of not-polyp images (dark bars in Figure 4). We draw attention to the lowest value obtained with the original model in the not-polyp test partition (from the original 0.88 to 0.85). Finally, in terms of the F1 score, the model trained with the highest number of not-polyp images showed the best F1 score in both test partitions (0.895 and 0.891).

### 3.3. Inter-Dataset Evaluation

Table 5 shows the performance of our five models, i.e., the baseline model presented in Nogueira et al. [11], plus the four new models trained with different percentages of not-polyp image injections, on the same public datasets analyzed in our previous work [12]. Globally, the average F1 score remains almost the same, with an F1 around 0.75 (see global averages in Table 5). As a result, we could discard an unexpected worsening in the public datasets.

However, when the testing results are divided into functions of whether the testing dataset contains not-polyp images or not, the results differ. In the case of the results obtained in the datasets that contain not-polyp images, the models trained with not-polyp images attain, on average, greater than or equal to the F1 scores of the original model, where the model with a 15% injection of not-polyp images is able to increase the F1 in the four datasets, raising the original average of 0.695 to 0.722. The behavior of the same models in the datasets that only contain polyp images is more heterogeneous, but a general decrease in the average F1 is observed, ranging from 0.749 to 0.799 in comparison with the original average of 0.800. 

**Figure 5:**
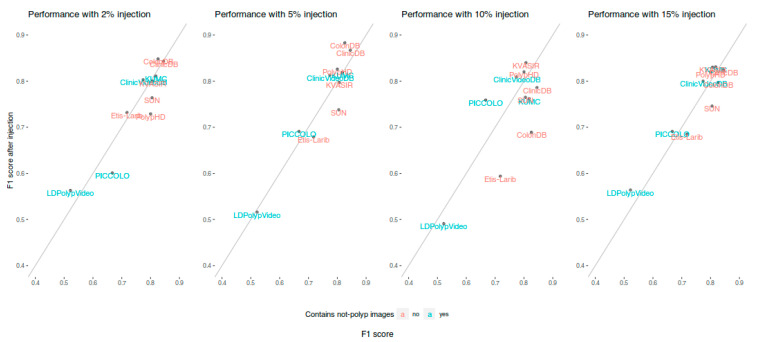
Comparison between the F1 performances obtained by our baseline model in the ten public datasets (X axis) and the F1 performances obtained by the four new models trained with not-polyp images (Y axis). Public datasets containing not-polyp images are marked in light blue. Points above the diagonal line represent benefit.

In order to visualize the performance changes among our four new models in each public dataset, Figure 5 compares the F1 performances obtained by our baseline model (X axis) and the F1 performances obtained by the four new models trained with not-polyp images (Y axis). The public datasets that contain not-polyp images are marked in light blue.

**Figure 6:**
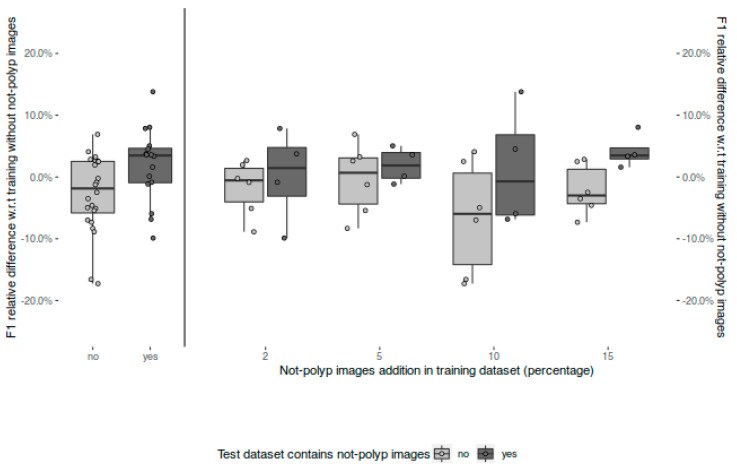
Relative F1 differences in the same test dataset with respect to our baseline model (trained without not-polyp images), and grouped test dataset type (i.e., containing or not containing not-polyp images).

Focusing more on the relative variations, Figure 6 shows the relative change on F1 among all the public datasets between the models trained with not-polyp images with respect to the original model (trained without not-polyp images) in the same test dataset.

Again, we have grouped the results according to the presence or not of not-polyp images in the test partition. The left part of Figure 6 shows a general benefit in the four public datasets with not-polyp images (dark boxes). By contrast, regarding the performance in the public datasets without not-polyp images, a general worsening is observed (light boxes). When the results are disaggregated by model, the right part of Figure 6 shows that the better performance is always obtained in datasets that include not-polyp images for all the models. Finally, the model trained with 15% of additional not-polyp images attains the highest benefit in the not-polyp-containing datasets (dark box in the right-most column in Figure 6), being the only one showing a benefit of all of the four public datasets with not-polyp images.

### 3.4. Qualitative Analysis

To perform a qualitative analysis, the five models (the base model presented in Nogueira et al. plus the new four models trained with different percentages of not-polyp image injections) have been used to process two videos at a rate of 25 fps: (i) an 18 s video of a polyp (450 frames); and (ii) a 14 s video of normal mucosa (not-polyp) (350 frames). The two videos are provided as Supplementary Videos S1 and S2, respectively. Table 6 shows a global summary of the predictions made over the two videos.

Regarding the polyp video, in Table 6 we see a general recall of 90%, in terms of distinct frames that contain at least one emitted bounding box, and very small differences among all the models (see relative change, third column in Table 6). Regarding the not-polyp video, we see a drastic reduction in the total number of bounding boxes, with respect to the original model, which were all false positives (see relative change, last column in Table 6).

**Figure 7:**
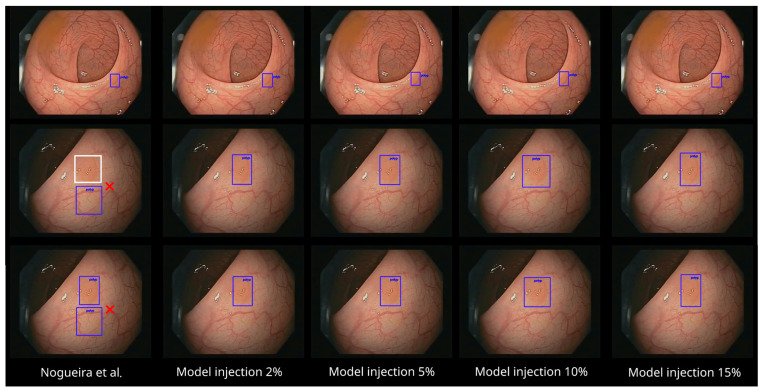
The five models with three sample frames of the polyp video analyzed (Supplementary Video S1). The false positive identifications are marked with red cross marks. The false negatives are marked in white boxes (added manually for illustration purposes) [11].

In order to give some illustrative examples within these two videos, Figure 7 contains some frames with bounding box predictions in the polyp video. As expected, the five models are able to detect the polyp correctly in almost all the frames (first row of Figure 7). However, in this specific polyp video, the new models even surpass the detection ability of the base model (slightly better recall, as shown in Table 6), where some of the false negatives (white box in the second row of Figure 7) in the original model were corrected by the not-polyp-trained models. Moreover, some false positive identifications (boxes not in the correct location of the polyp) emitted by the baseline model disappear in the not-polyp-trained models (second and third rows of Figure 7).

**Figure 8:**
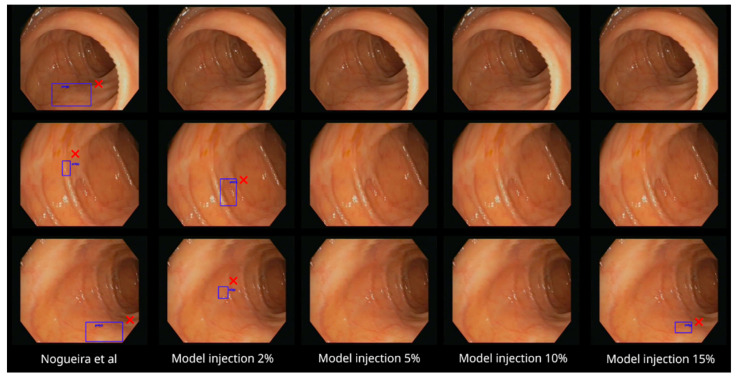
The five models with three sample frames of the not-polyp video segment analyzed (Supplementary Video S2). While the baseline model makes incorrect predictions (red cross marks) in the three cases, most of the not-polyp-trained models do not [11].

Regarding the not-polyp video, Figure 8 again shows three example frames. The base model produces false positive identifications in the three frames, while most of the not-polyp-trained models do not.

**Figure 9:**
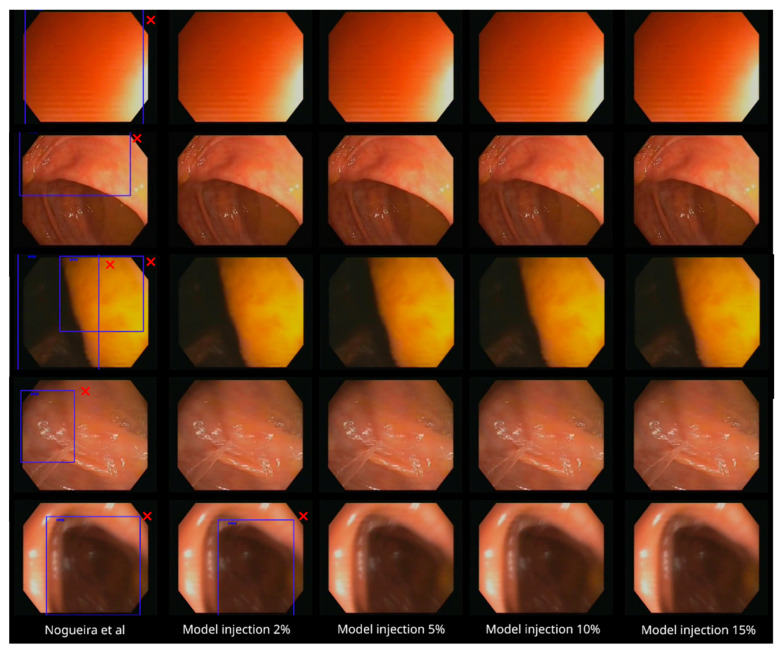
The five models facing different situations found in two exploration videos included in PIBAdb (provided as Supplementary Videos S3 and S4), including close to the wall of the colon (first row), water jet (fourth row), or blurry images due to water jet (fifth row). False positives are marked as red cross marks [11].

Focusing on the real out-of-distribution samples that we wanted to address in this study, we include two additional video clips found in two exploration videos. Figure 9 shows some frames of different situations found in two exploration videos included in the PIBAdb (provided as Supplementary Videos S3 and S4). These include, for instance, close to the wall of the colon images (first row), water jet (fourth row), or blurry images due to water (fifth row). The base model tends to produce false positives in such situations, as the first column shows, that the not-polyp-trained models tend to eliminate.

## 4. Conclusions

In this work, we retrained our polyp detection model with negative samples that were out-of-distribution images for our previous model in order to improve its positive predictive value (precision), while maintaining its sensitivity (recall), in the real setting. We have trained several models with different amounts of not-polyp images, incrementing our original training set from 2% up to 15% not-polyp images. We have evaluated them with both internal and public test partitions that include different amounts of such not-polyp images.

Regarding the performance of the models in our test partitions, or intra-dataset evaluation, we have observed that precision always increases as more not-polyp images are used for training the models, while recall oscillates slightly among them. The model trained with a 15% injection of not-polyp images was able to maintain the recall and reach the best precision of all the test partitions that include not-polyp images (average F1 of 0.893). Moreover, the original model was unable to maintain its original performance in the test partition that included the not-polyp images (descending from 0.881 to an average of 0.869), which illustrates the need for a test partition that includes this kind of image, which was kept out-of-distribution during the initial development.

On the other hand, we observed that the performance of the models in the public datasets containing not-polyp images also increases as more not-polyp images were used for training. Again, the model trained with a 15% injection of not-polyp images attained the best average F1 (0.722), and the biggest difference from the baseline model (average F1 of 0.695). In the public datasets with only polyp images, the results are more heterogeneous and differ depending on the dataset and model used. Altogether, the average F1 of each new model remained almost the same (~0.75), and, therefore, an unexpected worsening in the public datasets due to training with not-polyp images is discarded.

As noted before, the general trend is that the latest public datasets available include images without polyps, and our results suggest that it is worth using them (i) as negative samples for training, as it improves the performance in scenarios closer to real colonoscopies, and (ii) to correctly estimate the real performance of the detection models. The benefits are even observed when testing in datasets that only contain polyp images, although with higher variability.

Based on these observations, we think that there is room for improvement in polyp detection models using more data. Thus, future work to improve our model will be data-centric, as well. First, as the best performing model was the one trained with the highest amount of not-polyp images, also maintaining a good recall, it seems that the ceiling has not been reached in terms of the best proportion of not-polyp images to inject. More experiments in this direction could give us interesting insights. Our PIBAdb database still contains both annotated polyp images and not-polyp images that were not used to train the models, and it would be interesting to see if using more training data with both polyp and not-polyp images can increase the performance. Also, a lot of public datasets are available, and it would be interesting to see if training with some images from them (maybe the most challenging ones) can improve the performance in both our dataset and in the other public datasets.

## Figures and Tables

**Table 1:** Summary of the ten public colonoscopy image datasets for polyp location including: the name and publication/s related with the dataset (Dataset), the year when the dataset was published (Year), the image resolution (Resolution), the number of images in the dataset (No. of images), the method used to provide image location (Ground Truth), and whether the dataset includes images without polyps and the percentage of images with polyps in case they are included (Without polyps?).

Dataset	Year	Resolution	No. of Images	Ground Truth	Without Polyps?
CVC-ColonDB [18,19]	2012	574 × 500	300	Mask	No
ETIS-Larib [20]	2014	1225 × 966	196	Mask	No
CVC-ClinicDB [17]	2015	384 × 288	612	Mask	No
CVC-ClinicVideoDB [21,22]	2017	384 × 288	11,954	Mask	Yes (16.14%)
CVC-PolypHD [18,19]	2018	1920 × 1080	56	Mask	No
Kvasir-SEG [16]	2020	332 × 487 to 1920 × 1072	1000	Bounding box and mask	No
PICCOLO [23]	2020	854 × 480, 1920 × 1080	3433	Mask	Yes (1.25%)
KUMC [14]	2021	Various resolutions	37,899	Bounding box	Yes (5.08%)
SUN [13]	2021	1240 × 1080	49,136	Bounding box	No *
LDPolypVideo [15]	2021	560 × 480	40,186	Bounding box	Yes (15.7%)

* The SUN dataset contains 109,554 frames without polyps that were not downloaded for our experiments.

**Table 2:** Performance metrics in each validation partition for the selected models.

	Confidence Score Threshold	Recall	Precision	$F_{1}$	AP
Nogueira et al. [11]	0.19	0.905	0.912	0.909	0.920
Model injection 2%	0.24	0.897	0.917	0.907	0.920
Model injection 5%	0.18	0.911	0.915	0.913	0.923
Model injection 10%	0.22	0.884	0.918	0.901	0.918
Model injection 15%	0.15	0.903	0.920	0.912	0.923

**Table 3:** Performance metrics of each model on the original test partition of Nogueira et al., which does not contain not-polyp images.

Training Dataset	Recall	Precision	$F_{1}$
Nogueira et al. [11]	0.872	0.890	0.881
Model injection 2%	0.867	0.894	0.880
Model injection 5%	0.875	0.902	0.888
Model injection 10%	0.850	0.904	0.876
Model injection 15%	0.880	0.910	0.895

**Table 4:** Precision metrics obtained with different models against different not-polyp injections into the original test partition.

Model	Test Partitions (Percentage of Not-Polyp Augmentation)			
	2%	5%	10%	15%
Nogueira et al. [11]	0.882	0.871	0.852	0.836
Model injection 2%	0.890	0.886	0.879	0.871
Model injection 5%	0.899	0.895	0.888	0.881
Model injection 10%	0.902	0.901	0.897	0.895
Model injection 15%	0.909	0.907	0.904	0.901

**Table 5:** F1 score of the five trained models in the 10 public datasets. Averages are provided for all datasets, datasets with both polyp and not-polyp images (4 datasets), and for datasets with only polyp images (6 datasets).

	Training Dataset				
	Nogueira et al. [11]	Not-Polyp Injection			
		2%	5%	10%	15%
Datasets with both polyp and not-polyp images					
LDPolypVideo	0.522	0.563	0.516	0.491	0.564
ClinicVideoDB	0.774	0.803	0.813	0.809	0.800
KUMC	0.818	0.811	0.819	0.762	0.831
PICCOLO	0.667	0.601	0.691	0.759	0.691
*average*	*0.695*	*0.694*	*0.710*	*0.705*	*0.722*
Datasets only with polyp images					
ClinicDB	0.845	0.843	0.867	0.786	0.824
ColonDB	0.826	0.848	0.883	0.689	0.797
SUN	0.805	0.764	0.738	0.765	0.746
KVASIR	0.807	0.800	0.797	0.840	0.830
Etis-Larib	0.718	0.732	0.679	0.594	0.685
PolypHD	0.800	0.729	0.826	0.820	0.820
*average*	*0.800*	*0.786*	*0.799*	*0.749*	*0.784*
*global average*	*0.758*	*0.749*	*0.763*	*0.731*	*0.759*

**Table 6:** Summary of the network predictions in both polyp and not-polyp videos. For the polyp video, the total number of distinct frames with at least one bounding box is reported, as a proxy of the recall (assuming that, at least one of the bounding boxes in the frame is placed over the polyp). For the not-polyp video, the total number of bounding boxes is reported, which are all false positives. Relative changes if these metrics are also reported among different models.

	Polyp Video (450 Frames)		Not-Polyp Video (350 Frames)	
	Frames with at Least One Bounding Box	Relative Change	Total Bounding Boxes (False Positives)	Relative Change
Nogueira et al. [11]	404 (89.78%)	-	90	-
Model injection 2%	418 (92.89%)	+3.47%	43	−52.22%
Model injection 5%	419 (93.11%)	+3.71%	24	−73.33%
Model injection 10%	416 (92.44%)	+2.97%	16	−82.22%
Model injection 15%	407 (90.44%)	+0.74%	63	−30.00%

## Data Availability

Publicly available datasets were analyzed in this study. Our not-polyp datasets are a subset of PIBAdb Colorectal Polyp Image Cohort, which can be requested here: https://www.iisgaliciasur.es/home/biobanco/cohorte-pibadb. Third-party datasets are available upon request to their owners.

## Supplementary Materials

The following supporting information can be downloaded at: https://www.mdpi.com/article/10.3390/diagnostics13050966/s1, Supplementary Video S1 (S1_Video_polyp.mp4): an 18 s video of a polyp (450 frames). Supplementary Video S2 (S2_Video_Not_Polyp.mp4): (ii) a 14 s video of normal mucosa (not-polyp) (350 frames). Supplementary Video S3 (S3_Exploration_1.mp4): a 15 s video of an exploration from PIBAdb. Supplementary Video S4 (S4_Exploration_2.mp4): a 15 s video of an exploration from PIBAdb.
